# A Wireless Magnetic Resonance Device for Optogenetic Applications in an Animal Model

**DOI:** 10.3390/s20205869

**Published:** 2020-10-16

**Authors:** Arthur C. Tsai, Andrew Chih Wei Huang, Ying Hao Yu, Chii-Shyang Kuo, Chih-Chan Hsu, Yeou San Lim, Bai Chuang Shyu

**Affiliations:** 1 Institute of Statistical Science, Academia Sinica, Taipei 11529, Taiwan; arthur@stat.sinica.edu.tw (A.C.T.); 106581021@cc.ncu.edu.tw (C.-S.K.); crisstiger@stat.sinica.edu.tw (C.-C.H.); 2 Department of Psychology, Fo Guang University, Yilan County 26247, Taiwan; chweihuang@mail.fgu.edu.tw (A.C.W.H.); D0733001@niu.edu.tw (Y.H.Y.); 108754002@nccu.edu.tw (Y.S.L.); 3 Institute of Biomedical Sciences, Academia Sinica, Taipei 1529, Taiwan

**Keywords:** wireless remote-controlled device, magnetic resonance, untethered optical stimulation, optogenetics, rat, mice

## Abstract

The currents of optical stimulation devices with tethered or untethered systems have various disadvantages, including optical fiber breakage, disrupted animal movements, heavy batteries carried on heads, and high-frequency electromagnetic impacts. Our novel wireless remote control was developed to address these issues. The novel wireless device uses a magnetic resonance technique to modify the deficits of the conventional magnetic induction or radio-frequency power sources. The present device emits a strong and steady electromagnetic power. It is cheaper than previous versions, and the receiver coil on its head is very light (≦ 1 g). For the present wireless remote-controlled device, the electromagnetic field’s range (i.e., +5 cm and −5 cm of the outside coil) is larger than the range for the magnetic induction and radio-frequency power sources. The present device controls animals’ behavior by the electromagnetic field’s effective range via photostimulation. The novel wireless remote-controlled device with a magnetic resonance technique can be applied in many behavioral tasks in mice and rats. To avoid the adverse effects of high radio frequency and to extend the electromagnetic field’s range, this novel technique serves as a helpful tool to modulate the neuronal activity of target neurons in specific brain areas for optogenetic experiments.

## 1. Introduction

### 1.1. Overview 

In the neuroscience field, the optogenetic approach combines three fields (genetics, virus transfection, and optics) to excite or inhibit neuronal circuit activity [1]. This approach is designed to microinject a promoter’s specific clone DNA sequences (e.g., Calcium-calmodulin-dependent protein kinase II (CaMKII)), a light-gated ion-channel protein (e.g., channelrhodopsin-2 and ChR2), and opsin/florescence molecules through a specific virus vector (e.g., adeno-associated virus) into the target brain area. Thus, specific neuron types are induced in virus transfections. These transfected neurons generate a novel light-gated ion channel in the neuronal membrane. An optical fiber is implanted in the targeted brain area to deliver light to excite the neurons. The laser, or an light-emitting diode (LED) light, turns on via the optical fiber to stimulate the specific type of neurons in the targeted brain area [2,3]. Thus, the photostimulation can modulate the function of specific neurons in the targeted brain area. Optical stimulation is generally divided into tethered and untethered formats. A tethered optical stimulation device controls animals’ behavior or responses with a remote laser through an optical-fiber line implanted in the targeted brain area [4]. The other style of untethered optical stimulation device uses a remote power source to manipulate the LED light implanted above the head to govern the behavioral response [4].

### 1.2. Tethered Optical Stimulations 

The tethered optical stimulation device connects to the targeted brain area through optical fibers implanted with surgical glues, cement, and external fixtures [5,6,7]. The device uses photostimulation to alter specific neurons and to drive neuronal activity in the targeted brain area, and thereby controlling the animals’ behaviors. With a similar behavioral pharmacology approach, it can also drive or change a specific neuron without affecting other neurons in a targeted brain area [8]. Thus, researchers can investigate how specific neurons relate to behaviors associated with neurological diseases [9], psychiatric disorders [10], and motor functions [6].

The tethered optical stimulation requires a connection with an optic fiber, but it does not require additional devices. Hence, it reduces setup costs and has been applied in most recent optogenetic studies. The tethered optical device has been studied in many behavioral tasks in vivo. For example, using the optogenetic approach, a microinjection of ChR2 into the ventral tegmental area can stimulate glutamatergic neurons to induce dopamine firings in the nucleus accumbens, leading to conditioned place preference and self-reinforcement behaviors [11]. Optogenetic photostimulation in the basal ganglia has been demonstrated to regulate Parkinson’s disease-like motor behavior [6]. Targeting excitatory neurons and CaMKII promoters in the motor cortex with ChR2 has been shown to modulate motor-cortex function in behaviors [5]. Using the tethered optical stimulation, photostimulating the hippocampus altered fear-based conditioning in the memory-recall phase [7]. 

The tethered optical stimulation device does, however, suffer from shortcomings [2,4]. For example, the tethered optical stimulation is inconvenient for handling animals and for recording their behaviors, because of the tethered lines [4]. In chronic-treatment experiments, the tethered optical stimulation interfered with measured behaviors in animals, and the risk of breaking optical fibers and cables is incremental [2]. The tethered optical-stimulation device is limited by the number of animals in a single experiment. In addition, it cannot prevent disruptions of animals’ movements [12]. Moreover, the tethered optical-stimulation device was not applied in the social interaction test [12], because the tethered device’s lines can become tangled and break when the tethered optical device is used in multiple measurements. Therefore, the tethered optical device cannot be used for multiple measurements that involve many animals in the same experiment. 

In conclusion, a tethered optical-stimulation device is often used in optogenetic experiments. However, its disadvantages include the inconvenience of handling it around animals, the risk of breaking optical fibers and cables, a limited number of animals in a single experiment, the disruption of animals’ movements, and its inutility to conduct the social interaction test. 

### 1.3. The Untethered Optical-Stimulation Device: Battery-Powered and Battery-Free with Wireless Remote-Control Formats

Untethered optical stimulation devices were developed to resolve the tethered optical-stimulation device’s shortcomings. To our knowledge, they can be classified as battery-powered or battery-free devices with wireless remote-control formats. The subsequent sections introduce their advantages, disadvantages, applications, and examples.

#### 1.3.1. Battery-Powered and Wireless Remote-Controlled Devices

Battery-powered untethered optical-stimulation devices use an infrared (IR) transmitter to induce IR transmissions and send photopulses to the IR receiver. The IR receiver on the battery-powered LED stimulator receives and decodes IR photopulses. Accordingly, the LED lights up to modulate the neuronal activity through the ChR2 light-gated ion channel for a specific type of neuron. The LED modulation then changes the movement behaviors. 

Several previous studies revealed the capacity of the untethered stimulation device to eliminate the disadvantages of the tethered stimulation device [13,14]. Accordingly, animals were able to move freely in behavioral testing and avoided tangling or breaking the head’s tethered line. Moreover, the battery-powered untethered optical device can measure chronic and longitudinal experiments. A previous study revealed that it was possible to apply a battery-powered device on the head to photostimulate the primary motor cortex and induce muscle twitches using Thy1-ChR2-YFP [13]. The device conveniently mounts a high-polymer block on a mouse’s head [13]. In another study, researchers used a miniature wireless neural stimulator with batteries on the animal’s head. They applied the wireless neural stimulation device, including the chip, batteries, and electrode sites, on a zebra finch’s head to control its behavior [14]. The untethered battery-powered optical stimulation device eliminates many of the disadvantages of the tethered optical stimulation device. However, it is still plagued by shortcomings. For example, the weight of heavy battery-powered devices may interfere with animals’ freedom of movement. 

#### 1.3.2. Battery-Free and Wirelessly Powered Remote-Controlled Device 

Battery-free and wirelessly powered remote-controlled devices use radio frequencies (RFs) for transmission [15]. When powered, the microcontroller automatically induces a radio module that contains an antenna and a radio chipset. The RF is transmitted to the antenna in the motherboard module. The antenna sends the transmission to the microcontroller and then on to the LED power supply. Finally, the LED power supply turns on LED lights in the optics module to control the optical stimulation, elicit neural hyperactivity via the excitatory ChR2 light-gated ion channel, and affect the animal’s movement.

Recently, some novel wireless remote-controlled devices without battery power have been developed [15]. This type of optical device has many advantages over battery-powered and wirelessly controlled devices. For example, it is a smaller device (<1 cm^3^) and weighs less (= 2 g). The power delivered to the LED ranges from 2 to 4.3 W. However, battery-free and wirelessly powered devices have disadvantages. Multiple LEDs mounted in the optical module on the head induce the dissipation of heat generated during operation. Based on previous data, mobile phone-induced ultra-high frequencies, ranging from 300 M to 3 G Hz are harmful to human health [16,17]. Accordingly, a higher radio frequency from the battery-free and wirelessly powered device creates a higher electromagnetic wave (i.e., 1.2 G Hz), which may impact health [4]. The same effect occurred with another novel wireless remote-controlled device that similarly produced a very high radio-frequency power source, thereby creating a much larger electromagnetic wave (i.e., 1.5 G Hz) [18]. Therefore, these two battery-free and wirelessly powered devices create higher electromagnetic frequencies and probably affect individuals’ health.

### 1.4. Principle and Rationale for Magnetic-Resonant Wireless Remote Control 

In electromagnetism’s early phase, efforts were made toward wireless power transfer, including in experiments by Nikola Tesla on microwave power transmission [19,20]. However, Tesla coils and microwave power transfer (discovered later) involved undesirably low efficiency and high radiactive loss due to its omnidirectional nature. The large electric fields and microwave radiation also present common safety concerns [21]. Therefore, this radioactive transfer approach to wireless communication or power transmission may not suit wireless optogenetic manipulation.

This study proposes an alternative approach of nonradioactive wireless power transfer using magnetic resonance for optogenetic applications in animal models. Energy is transferred over the strongly coupled regime between two conductive loops, enabling efficient power transfer [22]. This power transfer differs from the usual nonresonant magnetic-induction method. Electromagnetic induction works because a source coil creates a magnetic field. Some of those field lines pass through the secondary coil to induce a current. In midrange transmission applications, the nonresonant induction method wastes much transmitted energy and becomes very inefficient. Our wireless device for optogenetic applications in animal models adopts a magnetic resonance coupling pursued by an MIT team that exploited some near-field electromagnetic coupling [22]. The wireless electromagnetic resonance device works by creating an electrical energy transfer between two coils, tuned to resonate at the same frequency. Based on electromagnetic coupling principles, a resonant-based electromagnetic field generator injects an oscillating current into a highly resonant source coil around the enclosure to create an oscillating electromagnetic field. The receiver coil on the animal head forming an LC circuit is designed to be small-sized and lightweight at the same frequency of magnetic resonance. It receives power from the electromagnetic field and converts it back into an electrical current that lights up an LED to stimulate the specific type of neuronal activity. Unlike in electromagnetic induction, receiving coils with capacitors on the end can be tuned to the transmitter frequency to improve power transmission efficiency by tunneling the magnetic field from the source to the receiver coil. This technology has been called nonradioactive power transfer and involves stationary fields around the coils rather than fields, whose energy spreads in all directions. Given that the electromagnetic waves tunnel, they do not propagate through the air to be absorbed or dissipated, eliminating the large energy-wasting problem. 

Therefore, the magnetic resonance wireless remote-controlled device was configured and applied in the various behavioral tasks described below.

## 2. Materials and Methods

### 2.1. Animals

Ten male Sprague-Dawley rats (300–400 g) and 10 male C57BL/6 mice (25–30 g) were housed in an air-conditioned room (21–23 °C, 50% humidity, and a 12 h light/dark cycle starting at 08:00 h) with free access to food and water. The performed experiments complied with the American Psychological Association’s ethical standards for animal treatment. All experiments followed the Academia Sinica Institutional Animal Care and Utilization Committee’s guidelines. Every effort was made to minimize animal suffering and to minimize the number of animals used.

### 2.2. Device 

The wireless remote-controlled device components include a plastic enclosure (outside edge: 25 cm; onside edge: 23 cm), an electromagnetic device with a source coil (5 turns), a flexible ground (diameter: 23 cm), an electromagnetic generator, a direct-current power supply (19 V), a relay device (HiLetgo, B00LW15A4W, Shenzhen, China), a stimulator (A-M Systems isolated pulse stimulator, Model 2000, Carlsborg, WA, USA), and a receiver coil (24 turns) associated with the LED light (wavelength: 470 nm; length: 5 mm; blue light, Onset-eo Company, New Taipei City, Taiwan). The device’s configuration and construction are detailed in the results section. 

### 2.3. Behavioral Tasks

#### 2.3.1. Open-Field Task: Motor Function and Anxiety Assessments

The open field was a circular plastic box with a 23-cm diameter. The central inside circle was 7 cm in diameter. The animals (mice and rats) were placed in the electromagnetic field. Their time spent outside the central circle indicated the magnitude of their anxiety via their behavior. In the open-field task, locomotion and anxiety behaviors were measured with video tracking software (Video Tracking Record System Version 1.17, SINGA Technology Corporation, Taipei, Taiwan). The task is suitable for mice and for rats.

#### 2.3.2. Zero-Maze Task

The zero-maze task was conducted on an annular dark-gray platform (60 cm in diameter). The outer range’s diameter was 17 cm. Quadrant lanes were 3 cm wide. The zero-maze task’s platform was constructed of foam board, divided into four equal quadrants. Two opposite quadrants were defined as the open arm. The remaining two quadrants were the closed arm. The quadrants were surrounded by opaque walls 15 cm high. The outer walls were constructed of dark-gray foam board, and the inner walls of black foam board. The task is suitable for mice but not for rats.

#### 2.3.3. Elevated Plus Maze 

The elevated plus maze task’s platform was also constructed of dark foam board and consisted of two open arms (3 cm wide and 21 cm long). Perpendicular to the open arms were two closed arms of the same dimensions with opaque walls and dark walls 15 cm high. The four arms met in a 3-cm center square region. The task is suitable for mice but not for rats.

#### 2.3.4. Forced-Swimming Test

The forced-swimming task design measures rodents’ depressive behavior. At the beginning of the experiments, each animal was placed individually in a plastic cylinder (23 cm around × 40 cm high), containing 25 cm of water (25 ± 1 °C). The water was filled to the source coil’s height, which induced electromagnetic power that affected the receiver coil on the animal’s head. In each session, rats were placed in water and forced to swim for 5 min. The time they floated, swam, and struggled was measured. Floating was defined as immobility, except for movements necessary to keep the head above water. Swimming behavior consisted of forward motion through the water with forepaws on the water’s surface. Struggling behavior describes an upright position in the water with forepaws breaking the water’s surface. A longer floating time with shorter swimming and struggling times indicated a stronger depression [23]. The task is suitable for mice and for rats.

#### 2.3.5. Conditioned Taste Aversion

Conditioned taste aversion is a type of Pavlovian conditioning. The animals could drink the saccharin solution (i.e., a conditioned stimulus, CS) and were then injected with an emetic drug, such as lithium chloride (i.e., an unconditioned stimulus, US). The conditioned taste aversion is designed to rely on a 25-mL burette with 0.1-mL graduations. The burette was attached to the inside wall of the plastic enclosure. In the present study, we only used data regarding consumption of the CS saccharin solution to determine the effect of conditioned taste-aversion learning. In general, the CS consumption decreased following a US injection, suggesting conditioned taste aversion learning. The task is suitable for mice and for rats.

#### 2.3.6. Tail Suspension Test

The tail suspension test was used to measure the strength of depressive behaviors. During the behavior tests, the animals were suspended by the tail from a ledge via adhesive tape (affixed approximately 1 cm from the tail’s tip). The distance between the tip of the animal’s tail and the floor was approximately 30 cm. The tail suspension test was designed to measure immobility behavior (defined as the absence of movement). The test lasted 6 min. During the testing phase, the animals’ immobility behavior was scored by a blinded experimenter. The task is suitable for mice and for rats.

#### 2.3.7. Social Interaction Test

For the social interaction test, pairs of rats either unfamiliar or familiar with each other were placed in the test arena. Then, the experimenter observed social interactions and overall motor activity for 5 min. For each pair of animals, the experimenter summed the total scores for each parameter of all behaviors. Social interaction time (seconds) was recorded, including time spent sniffing partners, climbing over and under partners, mutual grooming, genital investigation, and following and walking around partners. However, aggressive behaviors, such as biting, boxing, and pulling each other, were not considered social interaction. Passive social contact (e.g., sitting or lying with body contact) was also not recognized as social interaction and was therefore excluded from the social interaction score [24,25]. The task may be suitable for mice and for rats.

## 3. Results

### 3.1. Wireless Remote-Controlled Device Components: Electromagnetic Device, Power, Relay Device, Stimulator, Receiver, and Electrical Circuitry

**Figure 1:**
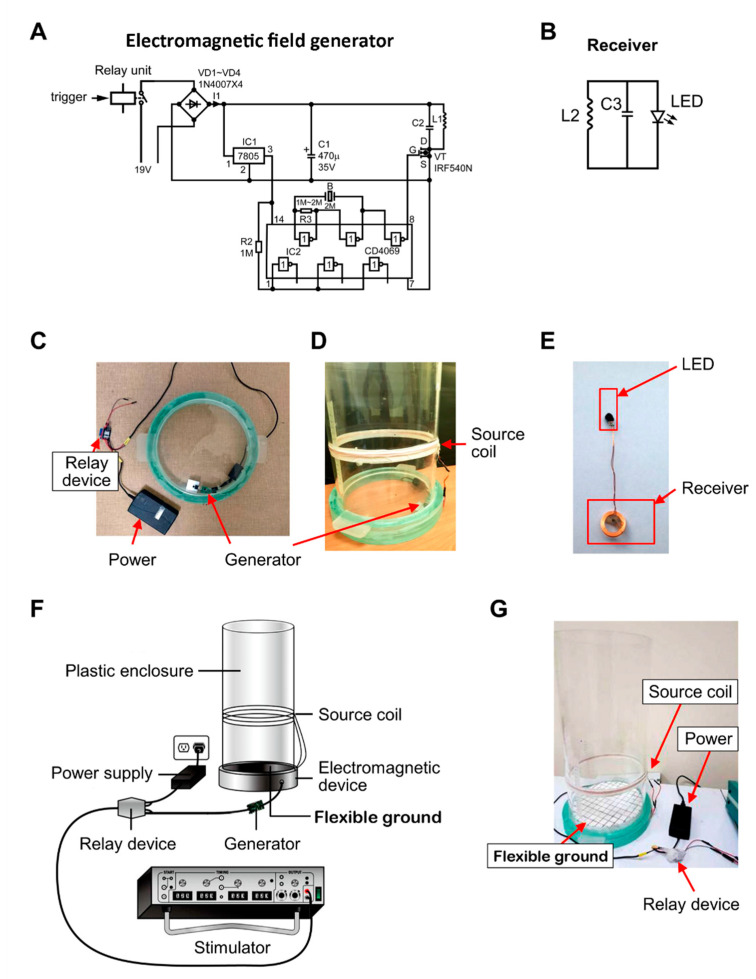
Description of the wireless remote-controlled device components. (**A**) The diagram of the electromagnetic field driving circuit. (**B**) The receiver circuit. (**C**) The power supply, relay device, and generator. (**D**) The source coil, generator, and plastic enclosure. (**E**) The receiver and the light-emitting diode (LED). (**F**) All the crucial components: plastic enclosure, electromagnetic device with a coil, flexible ground, power, relay device, stimulator, and a receiver associated with an LED light. (**G**) The electromagnetic field generator under the ground of the plastic enclosure.

The wireless remote-controlled device’s core components comprise the electromagnetic field generator and the receiver coil. Diagrams of the electromagnetic field-driving circuit and receiver are shown in Figure 1A,B. The driving circuit is based on the design of the electromagnetic resonator circuit for wireless charging systems [22,26]. The resonator circuit consists of a complementary metal–oxide–semiconductor (CMOS) Hex Inverter (CD4069, Texas Instruments Inc.), a power amplifier circuit (19 to 24 V), fixed-voltage LM7805 regulators, and an IRF540N MOSFET transistor (Figure 1A). In Figure 1C, the source coil consists of handmade helices of 5-turn copper loops with a radius of 25 cm and a 20 nF multilayer ceramic capacitor (MLCC), which is part of the driving circuit and creates a 200 KHz sine wave. The generator is designed to link the source coil with a line of positive charges, as well as with a line of negative charges to generate magnetic resonance (Figure 1C,D). The electromagnetic generator is located on the side opposite from the electromagnetic ground to avoid disrupting animals’ freedom of movement (Figure 1C,D).

Figure 1B shows the receiver circuit, with a small coil connected to a resonance capacitor that receives transmitted power based on a strongly magnetically resonant coupling between source and receiver coils, according to the coupled mode theory. The receiver coil is small enough to be portable and wirelessly powered by the source coil. Figure 1B shows that the receiver circuit comprised of a helical coil with a 1.5-cm radius, 24 turns of conducting wire, an SMD 0805 12nF MLCC, and a blue LED. 

The magnetic resonant frequency (in hertz) of the electromagnetic field generator and the receiver forming LC circuits is $f=1/\left(2\;\pi \sqrt{LC}\right)$, where L is the inductance in henrys, and C is the capacitance in farads. The receiver is attached to the backs of animals, and the LED light is implanted in specific brain areas targeted for stimulation or inhibition. When the power is on, the coil produces the electromagnetic field. Subsequently, the LED is lit. The neurons in the specific brain area are silent or active, thereby changing the animals’ behaviors (Figure 1E). 

Figure 1F depicts, in a schematic diagram, all components of the wireless remote-controlled device, and Figure 1G shows the constructed components. The power was switched on and off and controlled by a stimulator capable of delivering TTL, plus various parameters (stimulation frequency, duration, and inter-stimulus intervals), to the relay device, which controls the electromagnetic field’s production in the coil (Figure 1F). The present wireless magnetic resonance device’s crucial components include a plastic enclosure, an electromagnetic device with a coil, a flexible ground, power, a relay device, a stimulator, and a receiver associated with the LED light (Figure 1E–G). The plastic enclosure has a flexible ground and a coil above the flexible ground. The animals’ behaviors were tested in the plastic enclosure and supported by a flexible ground with an adjustable height (to enable the animal’s exposure to various electromagnetic fields). The electromagnetic field generator under the flexible ground resonantly transmits magnetic power into the receiver at the animals’ backs. The receiver’s LED is turned on to control neurons’ activity in a specific brain area (Figure 1G).

### 3.2. Parameter Tests for Receivers, Magnetic Resonance Field, and Wireless Photostimulating Remote-Controlled Devices

**Figure 2:**
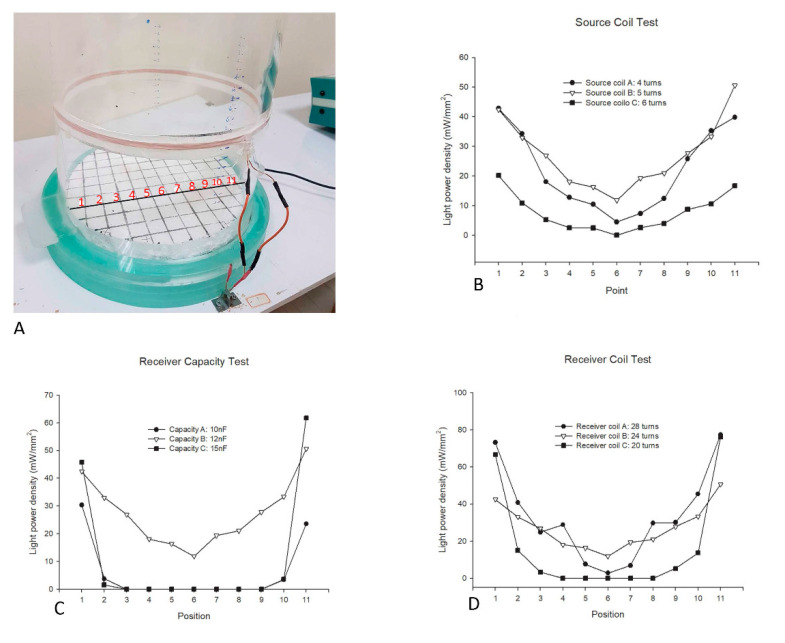
The wireless magnetic resonance device’s parameters. (**A**) The device’s light power density at the different points of a flexible ground (from 1–11) were tested. (**B**) Different turns of the source coil. (**C**) Different receiver capacities. (**D**) Different turns of the receiver coil.

The power density produced by the present wireless magnetic resonance device was tested at 11 points along a line parallel to the coil (Figure 2A). Although the resonance frequency (200-KHz) can be achieved via precise planning for turns of coil and capacity, we ran practical tests on various source coil turns (Figure 2B), receiver capacities (Figure 2C), and receiver coil turns (Figure 2D) to fine-tune the values of the generator and receiver circuits that contained L and C elements. Figure 2A depicts points 1–11 for light-power density measurements in the parametric testing. Points 1–11 were tested in the same plane as the source coil. The results showed that the 5-turn source-coil group had the greatest light-power density at points 1–11 compared to the other groups, including the 4-turn and 6-turn source-coil groups (Figure 2B). The capacity-B group exhibited a higher light-power density (12 nF) at positions 1–11 than the capacity-A group (10 nF) and the capacity-C group (15 nF) (Figure 2C). The receiver coil test indicated that the receiver-coil B group with 24 turns had the greatest light-power density, even relative to the 28-turn and 20-turn groups at points 1–11 (Figure 2D). Therefore, the tested parameters comprised multiple points on the flexible ground, source coil turn numbers, various receiver capacities, and receiver coils with various numbers of turns. The present study’s results suggest that most parameters that induced the highest light-power density were source coils with 5 turns (12-nF capacity) and receiver coils with 24 turns. Thus, the power values of the most parameter as mentioned were higher than 10 mW/mm^2^. In the experiments, the coil receiver was held on the head with sutures on the scalp, and animals moved freely under the wireless resonance control.

### 3.3. Electromagnetic Field Tested with Color-Coded Mapping 

**Figure 3:**
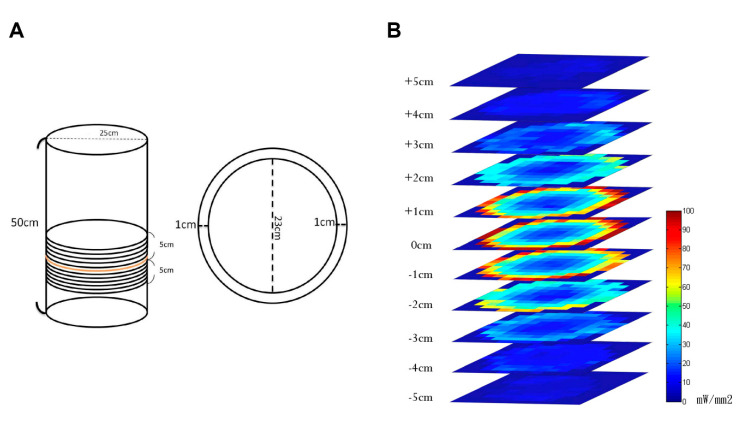
Color mapping for the electromagnetic field. (**A**) The electromagnetic field was composed of a plastic enclosure, a source coil, and a flexible ground. (**B**) For the electromagnetic resonance of the wireless remote-controlled device, the electromagnetic field is shown via color mapping.

The electromagnetic field’s strength was measured in a plastic enclosure with a flexible ground. The plastic enclosure’s outside edge had a diameter of 25 cm, and the inside edge had a diameter of 23 cm. The plastic enclosure was 2 cm thick. The plastic enclosure’s total height was 50 cm (Figure 3A). 

The electromagnetic field’s strength was systematically measured via the laser power meter (Laser Check, Michigan, MI, USA) and then color coded. Higher magnetic power was coded red, and the weaker magnetic power was coded blue. A blue LED light had a light wave of 470 nm and was used for effective power delivery. Every 1 cm, we recorded the blue light’s power as above or below the coil. The flat surface’s magnetic field was equally divided into approximately 132 small round areas. Each one was tested three times for 5–7 s. Later, these three values were averaged to determine the round areas’ light power. The power values were color coded as blue or red from lowest (0 mW/mm^2^) to highest (100 mW/mm^2^). The magnetic power’s available range was between +5 cm and −5 cm. A power value of approximately 1 mW/mm^2^ was necessary to stimulate neural activity with the LED [27,28,29,30]. Following the Kubelk–Munk model [T = 1/(Sz + 1)] [5], S is 11.2 mm^-1^ for a mouse and z is 40 μm, resulting in a transmission fraction (T) of 69.06%. S is the scatter coefficient per unit thickness and z is the thickness of the sample [5]. The power vale produced by the adjusted parameter was at least 10 mW/mm^2^. Thus, after 69.06% transmission fraction, the power values of the present device could produce at least 6.906 mW/mm^2^. Some researchers have reported that power values less than 0.1 mW/mm^2^ could induce ChR2 virus-transfection neural activity following photostimulation with the blue LED [31,32] (Figure 3B). Thus, taking the light attenuation into account, the power value of the device was still higher than the threshold power value (0.1 mW/mm^2^). The results showed the effective magnetic strength distributed throughout heights between +5 cm and −5 cm, which is suitable for behavioral measurement (Figure 3B). 

### 3.4. Auto-Tracking the Receiver on the Brain for Locomotor Activity in the Magnetic Resonance Field

**Figure 4:**
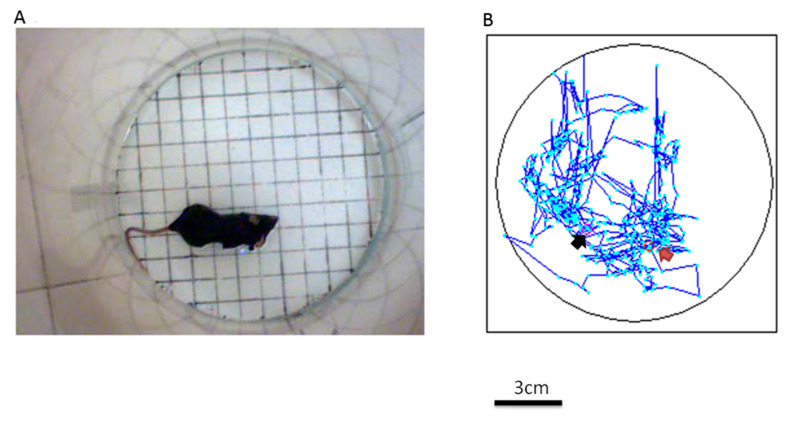
Auto-tracking device for testing motor functions in an electromagnetic field test. (**A**) The animal is placed in the electromagnetic field, and an auto-tracking device measures locomotive activity via programming to catch the blue light on the head of the mouse in the electromagnetic resonance field. (**B**) The traveled path is drawn with a blue line in the electromagnetic field. The black arrow represents the starting point and the red arrow represents the ending point.

The mouse was placed in the electromagnetic field, and locomotor activity was measured via an auto-tracking device programmed to capture the blue light on the mouse’s head in the electromagnetic resonance field (Figure 4A). The mouse’s distance traveled was recorded during the testing periods. Its path in the electromagnetic field was drawn with a blue line, and it was possible to analyze it and to permit a comparison of the control and experiment groups via the video tracking software (Video Tracking Record System Version 1.17, SINGA Technology Corporation, Taipei, Taiwan; Figure 4B).

### 3.5. Open-Field Task and Zero Maze Used to Test Locomotor Activity and Anxiety 

**Figure 5:**
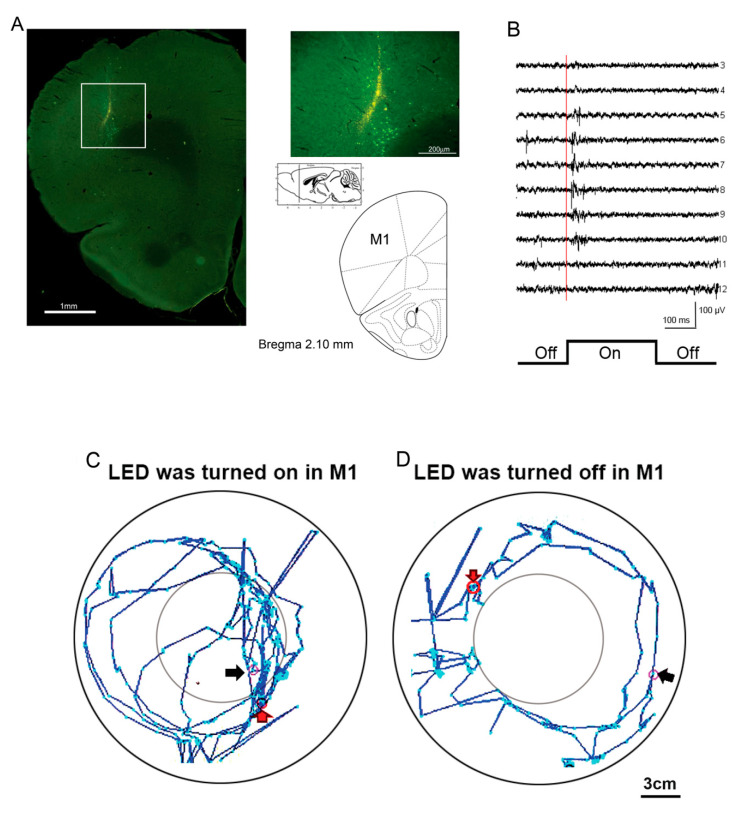
Optogenetic photostimulation outcomes to test locomotor activity in the open field task. (**A**) The Channelrhodopsin-2 (ChR2) transfection in the primary motor cortex (M1). Left: The whole view of the brain section. Right: The enlarged portion of the square on the left and the atlas show the relative section in sagittal and coronal views, +2.10 mm rostral to bregma. (**B**) An example of multichannel neuronal unit activity recording in the M1. The marker indicated the turning on of the optogentic stimulation. (**C**) The picture shows the LED light turned on in M1, the primary motor cortex. (**D**) The picture shows the LED light turned off in M1, the primary motor cortex. The black arrow represents the starting point and the red arrow represents the ending point.

**Figure 6:**
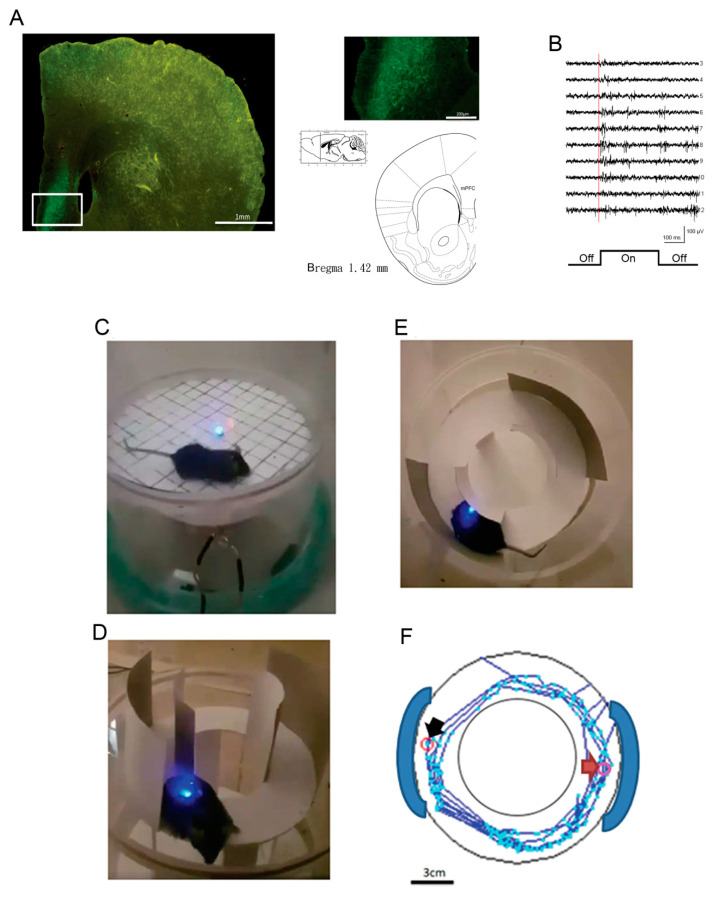
Anxiety behavior tests in the zero-maze and open-field tasks with optogenetic photostimulation. (**A**) The ChR2 transfection in the medial prefrontal cortex (mPFC). Left: The whole view of the brain section. Right: The enlarged portion of the square on the left and the atlas show the relative section in sagittal and coronal views, +1.42 mm rostral to bregma. (**B**). An example of multichannel neuronal unit activity recording in the mPFC. The marker indicated the turning on of the optogentic stimulation. (**C**–**E**) These pictures depict testing anxiety behavior in the zero-maze task; the optical fiber is implanted into the mPFC. (**F**) Using an auto-tracking device, the animal’s path was drawn using photostimulations into the mPFC in the zero-maze task. The black arrow represents the starting point and the red arrow represents the ending point.

It was possible to conduct the open-field task to test motor functions and measure anxiety behavior. It appropriately consisted of a circle with a 10-cm diameter in the middle of the magnetic field. When an animal entered the center circle, the cross numbers and the time it spent there were recorded. Higher cross numbers and more time indicated high-anxiety responses. In the magnetic field, the animals were able to move freely and were controlled by the photostimulation of the blue LED light. The microinjection was 0.3~0.4 μL volume of ChR2 AAV virus [AAV-CaMKIIa-hChR2(H134R)-EYFP] into the target site and the glutamate neurons were transfected with the virus. The stimulus frequency was defined as 20 Hz, duration was 50 ms, and the inter-stimulus interval was 25 ms. The animal with a ChR2 virus transfection had an optical fiber implanted in its primary motor cortex (M1), associated with the receiver on the head. The ChR2 transfection site in the M1 is shown in Figure 5A. The M1 has been shown to mediate the motor function [33], and the photostimulation with ChR2 virus transfection modulated the motor activity in the open field task. When the power supply was turned on, the coil produced an electromagnetic range of +5 cm and -5 cm. For mice, the distance between the flexible ground and the plastic enclosure’s coil was 6 cm, so the receiver was within the range for optimal optogenetic stimulation. Later, the mice with ChR2 transfection exhibited hyperlocomotion. When the LED was turned on, the mice’s max speed increased (234.09 cm/s), and they traveled a longer distance (82.874 cm). When the LED was turned off, the max speed decreased (87.29 cm/s), as did the total distance traveled (48.244 cm). For rats, it was appropriate to enlarge the distance between the flexible ground and the coil to 13.5 cm for optimal optogenetic stimulation. The animal’s locomotor activity in the open-field task was video recorded when the LED was turned on or off (Figure 5B,C). The animals changed locomotor activity in the open field test. To analyze anxiety behavior in the open-field test, the optical fiber was implanted into the medial prefrontal cortex (mPFC), which was associated with anxiety and fear behaviors [34]. The magnetic power was transferred to the receiver coil and controlled by the relay device and stimulator. The optical fiber transferred signals to excite the mPFC’s neurons and showed hyperactivity through photostimulation. Figure 6A shows the ChR2 transfection site in the mPFC. The animals showed a change in cross numbers and spent time in the center square, indicating anxiety behaviors (Figure 6B). On the other hand, the zero-maze task can also test anxiety behaviors. In this zero-maze task, the animals showed entries and spent time in the open arm (Figure 6C) and in the closed arm (Figure 6D), indicating anxiety. The cartoon picture depicts the animals’ routes between the open arms and the closed arms in the zero-maze task (Figure 6E). In Figure 6E, the black arrow represents the starting point and the red arrow represents the ending point. 

### 3.6. Elevated Plus Maze Task for Anxiety

**Figure 7:**
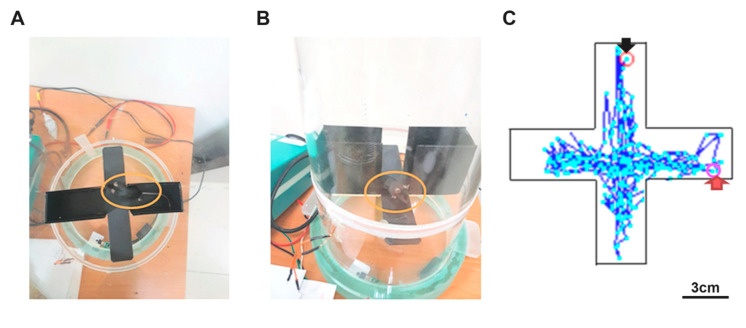
Anxiety behavior test in the elevated plus maze task with optogenetic photostimulation. (**A**,**B**) These pictures depict testing anxiety behavior of the elevated plus maze task; the optical fiber was implanted into the medial prefrontal cortex (mPFC). (**C**) Using the auto-tracking device, the animals’ path was drawn with photostimulations into the mPFC in the elevated plus maze task. The black arrow represents the starting point and the red arrow represents the ending point.

The elevated plus maze task is another anxiety behavioral test. During this task, the mice or rats had to wear the receiver on their heads, and the associated optical fiber had to be implanted in the mPFC. The electromagnetic power was triggered, and the receiver transferred photostimulation to the mPFC to produce anxiety responses. Fewer animals entered the open arm, and animals spent less time in the open arm (Figure 7A). The orange circle indicates the mouse’s location (Figure 7B). The cartoon picture depicts the route that animals traveled between the open arms and closed arms in the elevated plus maze task (Figure 7C). In Figure 7C, the black arrow represents the starting point and the red arrow represents the ending point.

### 3.7. Forced-Swimming Test for Depression

**Figure 8:**
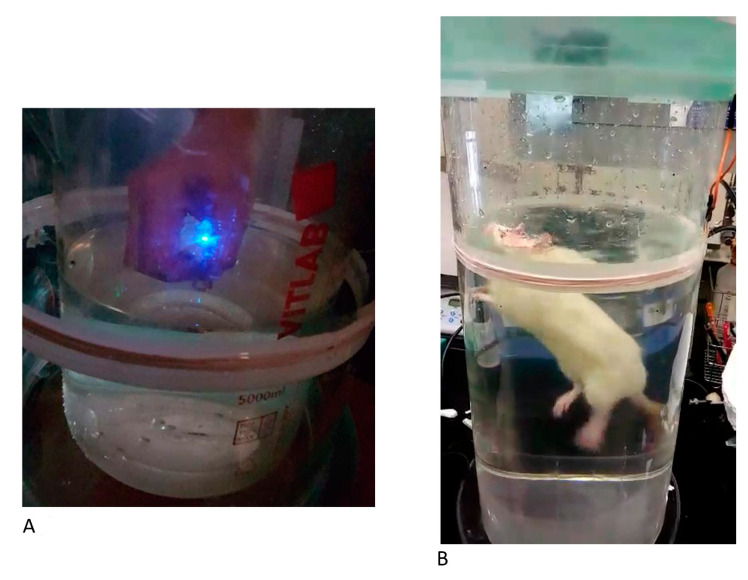
Depression behavior test in the forced-swimming test with optogenetic photostimulation. (**A**,**B**) These images depict depression behavior testing during the forced-swimming test; the optical fiber was implanted into the medial prefrontal cortex (mPFC).

The forced-swimming test is a typical assessment of depression in animals. Each given rodent had an optical fiber implanted in the mPFC, and the optical fiber is linked with the receiver. The animals were transfected with an excitatory ChR2 virus in the mPFC. Triggering the power supply turns on the electromagnetic power in the magnetic field. The magnetic power delivers the photostimulation to the mPFC. The animals with the ChR2 virus transfection were controlled by the wireless magnetic resonance device and showed increased depressive behavior. The rodents spent more time floating and less time swimming and struggling, indicating increased depressive behaviors. Figure 8A shows that the LED was turned on when the receiver coil was placed on the magnetic field’s edge. To test depressive behaviors, the rat was placed in the cylindrical tank for the forced-swimming task.

### 3.8. Conditioned Taste Aversion Learning

**Figure 9:**
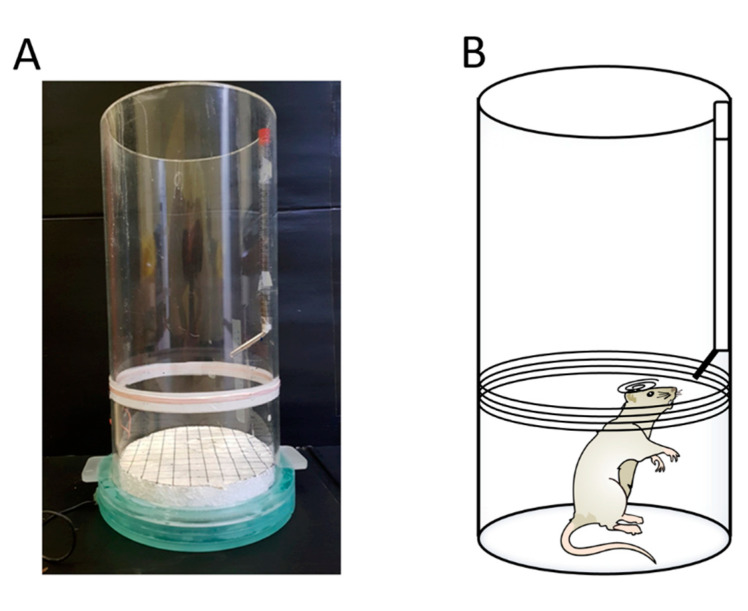
Conditioned taste aversion test with optogenetic photostimulation. (**A**) The wireless remote-controlled device was applied in the conditioned taste aversion learning task; a drinking tube was linked to the enclosed wall of the wireless remote-controlled device. (**B**) An illustration showing an animal with photostimulation licking the drinking fluid tube after conditioned taste aversion.

In the electromagnetic field condition, the rodent placed in the electromagnetic field had to have an untethered optical fiber implanted in its mPFC. The fiber was linked to the receiver, which was placed on the animal’s back. The receiver produced magnetic power from the coil in the plastic enclosure, and the magnetic power controls the photostimulation in the brain to increase the mPFC’sneural activity. Later, the mice or rats drank more CS solution, indicating a smaller conditioned taste aversion learning effect (Figure 9A). Figure 9B depicts mice drinking water or the CS solution in the electromagnetic field.

### 3.9. Tail Suspension Test

**Figure 10:**
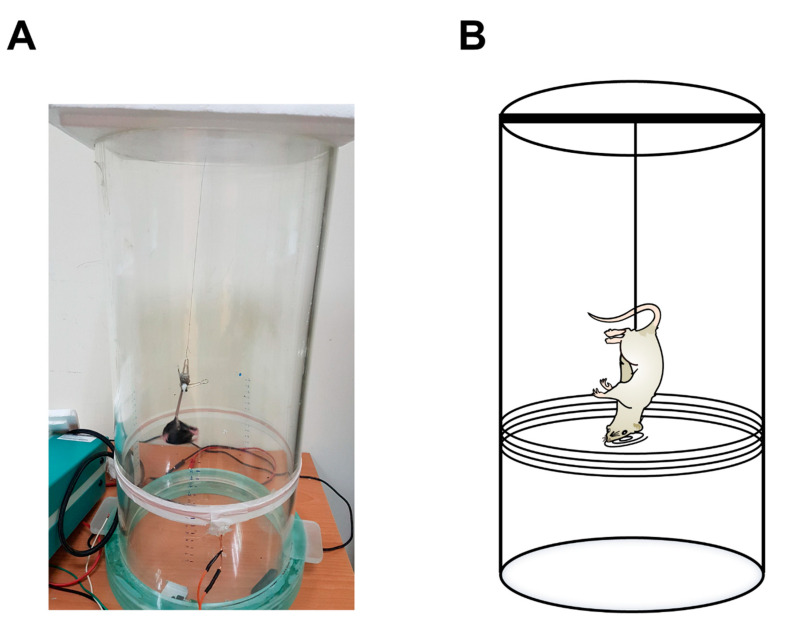
Depression test in the tail suspension task with optogenetic photostimulation. (**A**) The wireless remote-controlled device was applied in the tail suspension task. To test depression behavior in the tail suspension test, the optical fiber was implanted into the medial prefrontal cortex (mPFC). (**B**) An illustration showing an animal with photostimulation to test depression in the tail suspension task.

The tail suspension test is designed to test depressive behavior in mice or in rats. The immobility time was recorded for the depression index. The rodent’s tail was attached to a pole, and its immobility time was measured. The very lengthy immobility time indicated a strong depression response. During the electromagnetic device’s conduction, the rodents had an optical fiber implanted in their mPFC. The optical fiber was associated with the receiver, which was placed on the rodent’s back. The electromagnetic power was triggered, inducing the magnetic power that is received by the receiver. The receiver controlled the optical fiber’s photostimulation to increase the mPFC’s neural activity. After the mPFC was excited, the rodent showed more mobility, indicating decreased depressive behaviors (Figure 10A). Figure 10B depicts the tail suspension test in the electromagnetic field.

### 3.10. Social Interaction Test

**Figure 11:**
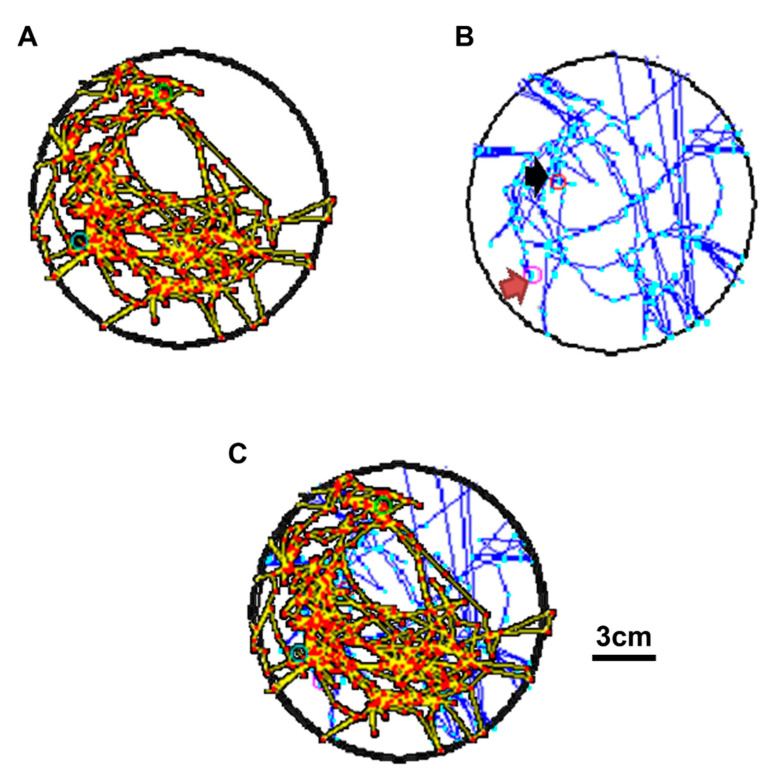
Social interaction test with optogenetic photostimulation. To test social interaction behavior, two animals were placed in the magnetic field, and the optical fiber was implanted into the medial prefrontal cortex (mPFC). (**A**,**B**) Using an auto-tracking device, the distances traveled by each animal are respectively drawn. (**C**) The picture combines the distances traveled by two animals with photostimulation in the social interaction test. The black arrow represents the starting point, and the red arrow represents the ending point.

During social interaction tests in the magnetic field, the rodent had an optical fiber implanted in the mPFC. The power supply was turned on, and the power was transmitted into the relay and stimulation to stably modulate the strength and frequency parameters. Following the power delivery, the electromagnetic field was created. The receiver on the animal’s back receives the magnetic power to stimulate the optical fiber in the animal’s brain and to increase the mPFC’s neural activity. Based on previous findings, exciting the mPFC enhances animals’ social interaction functions [35]. Photostimulating the mPFC decreased social interaction behaviors via the wireless magnetic resonance device. Regarding the social interaction test, the first animal moved around in the electromagnetic field (Figure 11A), and another animal moved around in the electromagnetic field, along with the first one (Figure 11B). To combine their routes, social interaction was measured via the schematic diagram of Figure 11C. 

### 3.11. An Example of Photo Stimulations for Testing Methamphetamine-Induced Conditioned Taste Aversion Learning

**Figure 12:**
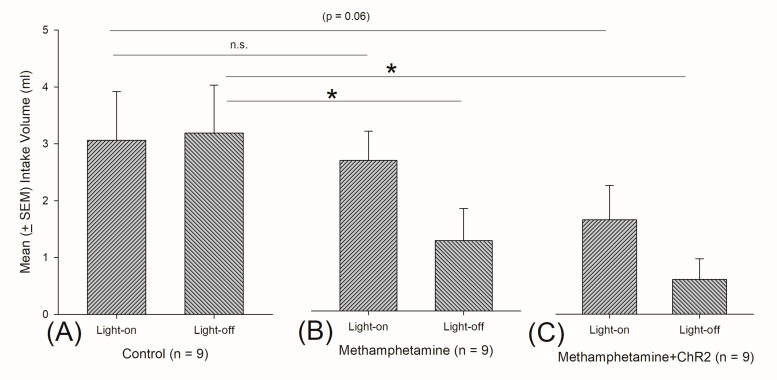
Methamphetamine-induced conditioned taste aversion learning test with ChR2 virus optogenetic photostimulation with LED light-on and light-off. To test conditioned taste aversion learning behavior, the optical fiber was implanted into the medial prefrontal cortex (mPFC). All rats were transfected with ChR2 virus or its control AAV-CaMKIIa-EYFP virus. Later, all of them were respectively assigned into (**A**) control (saline), (**B**) methamphetamine, and (**C**) methamphetamine + ChR2 three groups. All rats were allowed to drink 0.1% saccharin solution for 3 min light-on and 3 min light-off, and then they were intraperitoneally injected with a saline or methamphetamine. The next day, the rats were given the same procedure for drinking 0.1% saccharin solution for 3 min light-on and 3 min light-off in the test phase. The intake volume of saccharin solution was measured for each rat. A dependent t-test was conducted for light-on and light-off in the control, methamphetamine, and methamphetamine-ChR2 groups. The intake volume of LED light-on and light-off was not significantly different for the control (t = −0.13, *p* > 0.05), methamphetamine (t = 1.87, *p* > 0.05), and methamphetamine + ChR2 (t = 1.76, *p* > 0.05). Furthermore, a 3 × 2 mixed two-way ANOVA was conducted and post hoc tests with light-emitting diode (LSD). To test conditioned taste aversion induced by methamphetamine in behavior, the intake volume of saccharin solution in LED light-on for the methamphetamine + ChR2 group seemingly facilitated conditioned taste aversion learning when compared to the control group in LED light-on (*p* = 0.06). The methamphetamine group in light-on did not show a significant difference for intake volume (*p* > 0.05). Therefore, the LED light-on-induced photostimulation in the mPFC probably enhanced conditioned taste aversion learning by methamphetamine.

To test conditioned taste aversion learning behavior, the optical fiber was implanted into the mPFC. Rats were transfected with ChR2 virus or its control AAV-CaMKIIa-EYFP virus. Later, all of them were respectively assigned into (A) control (saline), (B) methamphetamine, and (C) methamphetamine + ChR2 three groups (Figure 12A–C). All rats were allowed to drink 0.1% saccharin solution for 3 min LED light-on and 3 min LED light-off, and then they were intraperitoneally injected with a saline or methamphetamine. The next day, the rats were given the same procedure for drinking 0.1% saccharin solution for 3 min LED light-on and 3 min LED light-off in the test phase. The intake volume of saccharin solution was measured for each rat. To test the intake volume of saccharin solution between the light-on and light-off in the control, methamphetamine, and methamphetamine + ChR2 groups, a dependent t-test was conducted. The intake volume of light-on and light-off did not have significant differences for the control (t = −0.13, *p* > 0.05; effect size = 0.1252), methamphetamine (t = 1.87, *p* > 0.05; effect size = 1.8697), or methamphetamine + ChR2 (t = 1.76, *p* > 0.05; effect size = 1.7611). The intake volume of light-on and light-off for each group did not show a significant difference, and the values of the effect size for each group were fewer than 0.2. Thus, they were lower than the values of the effect size for the control, methamphetamine, and methamphetamine + ChR2 groups. Actually, the intake volume of saccharin solution for light-on and light-off in the methamphetamine and methamphetamine + ChR2 groups might attain the ceiling effect, and they could not dissociate the significant effect between light-on and light off in the methamphetamine and methamphetamine + ChR2 groups. Alternatively, for testing of the conditioned taste aversion induced by methamphetamine in behavior, a 3 × 2 (group vs. light) mixed two-way ANOVA was analyzed for the intake volume of saccharin solution. The factor of group was a significant difference [F (2, 24) = 6.19, *p* < 0.05; partial η^2^ = 0.340]. Non-significant differences occurred in light [F (1, 24) = 1.97, *p* > 0.05; partial η^2^ = 0.076] and group x light [F (2, 24) = 0.76, *p* > 0.05; partial η^2^ = 0.060]. The particle η^2^ of group, light, and group x light were respectively higher than 0.059 or 0.138; thus, the manipulations of these three factors had good values of effect size. Post hoc tests with Fisher’s least significant difference (LSD) were conducted for the control, methamphetamine, and methamphetamine + ChR2 groups in light-on or light-off conditions. Light-on for the methamphetamine + ChR2 group seemingly facilitated conditioned taste aversion learning when compared to the control group in light-on (*p* = 0.06). However, the methamphetamine group in light-on did not show a significant difference for intake volume when compared to the control with light-on (*p* > 0.05). However, comparing light-off data for the control, methamphetamine, and methamphetamine + ChR2 groups, the intake volume of saccharin solution for the control group was significantly different compared to that of the methamphetamine (*p* < 0.05) and the methamphetamine + ChR2 groups (*p* < 0.05). Therefore, the LED light-on induced photostimulation in the mPFC probably enhanced conditioned taste aversion learning induced by methamphetamine (Figure 12).

## 4. Discussion

### 4.1. Optogenetic Photostimulation Outcomes: Wireless Control of Behaviors in Various Behavioral Tasks 

The present wireless magnetic resonance remote-controlled device can be used on rodents in numerous behavioral tests. For example, it can measure motor functions in locomotive activities in the open-field task. It can also test anxiety in the open-field test when the rodent’s route takes it to the field’s center. The zero-maze task, on the other hand, measures behavioral responses. The forced-swimming test concerns depressive behavior. A drinking tube was attached to the inside of the plastic enclosure. It was possible to record the amount of the taste fluid a rodent drank in the magnetic field to measure taste-aversion conditioning. In the tail suspension test, the animals were hung upside down above the plastic enclosure by their tails to test depressive responses. Moreover, it was possible to conduct social interaction tests in the magnetic field to test social behavior. The electromagnetic field is controlled by the power, and the magnetic resonance delivers the magnetic power into the receiver implanted in the rodent’s head. The receiver triggers the LED light’s photostimulation, thereby changing the neural activity of the brain areas targeted by the implanted untethered optical fiber. Finally, the tested behavior was controlled via photostimulation. The photostimulation’s frequency was 20 Hz in each period, and the power supply was 19 V, 3.42 A, and 65 W. Each behavioral task is detailed as follows. 

### 4.2. The Novel Wireless Remote Control: Its Applications and Advantages Compared to Other Devices

The present novel wireless remote-controlled device uses the magnetic resonance method referred to in the previous literature [22]. The present wireless remote-controlled device replaces electromagnetic induction and radio-frequency power source techniques. In our device, electrical power transforms to induce a magnetic field via a coil receiver on the rodent’s head to receive the magnetic resonance power from the outside source coil, thereby controlling the LED photostimulation to modulate the specific type of neuron and drive neuronal activity within the targeted brain area. Our wireless remote-controlled device has many advantages. First, the electromagnetic field is stronger, even compared to the electromagnetic induction or radio-frequency power source techniques. Second, less power is required from the power supply (approximately 19 V), and it produces a lower electromagnetic wave. The lower electromagnetic wave is much safer for individuals. Third, the equipment’s total price is cheaper. Fourth, the present device is wireless, without a tethered optical stimulation. Fifth, the receiver coil on the head does not need a battery; thus, the head coil is lightweight (≦1 g). Sixth, the present wireless remote-controlled device can be applied in various behavioral tasks. Seventh, the present device is suitable for mice and for rats. Eighth, the magnetic field’s range (+5 cm~−5 cm, based on the outside coil) is greater than that of magnetic induction or radio-frequency power sources. For example, we found that the magnetic induction’s range was approximately 1 cm~3 cm above the outside coil in our test. Moreover, the magnetic field’s scope with the magnetic induction or radio-frequency power sources had more variance compared to the present wireless remote-controlled device using the magnetic resonance technique. The floor was also designed to be flexible, and it was possible to adjust the floor’s height for various behavioral tasks. Thus, it was possible to control the tested animals by the wireless remote-controlled device via the magnetic field. Based on the present device, this study demonstrated that the wireless remote-controlled device, using a magnetic resonance technique, triggers photostimulation and controls neuronal activity within the brain during various behavioral tasks. Altogether, we compared tethered and untethered optical stimulation devices, and Table 1 describes their advantages and disadvantages.

### 4.3. Limitations of the Present Wireless Remote-Controlled Device

In the present wireless remote-controlled device, some limitations exist. For example, the electromagnetic field’s range is restricted to between +5 cm and −5 cm from the source coil. Thus, the present wireless remote-controlled device does not work if the animal moves outside that range. In addition, the inner space of the wireless remote-controlled device is very small, so the present wireless remote-controlled device is more suitable for mice. Thus, some behavioral tests—such as social interaction, elevated plus maze, and zero maze tests—are not suitable for measuring rats’ behaviors. Finally, the source coil and the receiver should be parallel to produce the most magnetic power. When the receiver coil is perpendicular with the source coil, the electromagnetic power disappears. Further modifications are intended to address the limitations.

## 5. Conclusions

The present wireless remote-controlled device, with a magnetic resonance technique, can be applied in many behavioral tasks of mice and rats. Moreover, the wireless remote-control device can drive the production of excitatory ChR2 photostimulation in specific neurons within a specific brain area to change behaviors. Furthermore, this device can induce an inhibitory ArchT photostimulation to change the tested behavior in another way with constant illumination at 1–5 mW/mm^2^ at the neuronal level [36]. Our wireless remote-controlled device has many advantages. For example, this novel device can avoid a high radio frequency and enhance the electromagnetic field’s range. The electromagnetic power is stronger and more even. Moreover, the wireless remote-controlled device uses less power. The receiver coil on the head is lighter and does not interfere with an animal’s behavior. The ground is flexible and can be adjusted to the animal’s height. This novel technique provides a significant and helpful tool to modulate neuronal activity in specific neurons within the selected brain areas for optogenetic experiments in neuroscience. As mentioned above, the advantages of the wireless remote-controlled device have improved the ability to implement such an approach in an average optogenetic lab. Moreover, all components of the wireless remote-controlled device could be manufactured as a tool kit and can be easily used and applied in the laboratory. The patent for the present wireless remote-controlled device is in process, and it is anticipated to enter the market in the near future. 

## 6. Patents

The current work’s patent has been applied for (#O104-18002US) and the patent is resulting from the work reported in this manuscript.

## Figures and Tables

**Table 1:** Comparison of the advantages and disadvantages between the tethered and untethered optical stimulation devices.

	Tethered Optical Stimulation	Untethered Optical Stimulation		
		Battery-Powered Device	Battery Free/Wireless-Powered Device	Our Wireless Magnetic Resonance Device
**Advantage**	Photostimulation via optical fiber to control neural activity in brain	Record the behaviors for behaving animals to avoid the tangled or breakage	Small device and light weight (= 2 g)	Even and stronger electromagnetic field
	2. Alter specific type of neurons to control behaving animals	2. Use the device in the chronic or longitudinal experiments	2. miniature for device on the head	2. Power supply is lower to induce a smaller electromagnetic wave
		3. No electromagnetic impact	3. Use a resonant radio-frequency power	3. The device is cheaper
				4. Wireless control
				5. Smaller device and light weight (receiver coil ≦ 1 g)
				6. Apply to measure various behaviors
				7. Apply for rats and mice
				8. The extent of the magnetic field is a wide range
**Disadvantage**	Inconveniently handling animals	Heavy battery powered device block behaving animal	Multiple LEDs mounted on the head to induce higher heat during operation	Less magnetic strengths when behaviors reach more than high of 10 cm.
	2. Easy breakage of optical fibers		2. High frequency electromagnetic impact (i.e., 1.2–1.5 × 10^9^ Hz)	
	3. Limit the number of animals at a single experiment			
			3. Uneven electromagnetic field	
	4. The device cannot prevent to disrupt animal movements			
	5. Social interaction testing problem			
